# Aliskiren-Loaded Nanoparticles Downregulate (Pro)renin Receptor and ACE Gene Expression in the Heart of Spontaneously Hypertensive Rats: Effect on NADPH Oxidase

**DOI:** 10.3390/ijms25020846

**Published:** 2024-01-10

**Authors:** Andrej Barta, Martina Cebova, Andrej Kovac, Martina Koneracka, Vlasta Zavisova, Olga Pechanova

**Affiliations:** 1 Institute of Normal and Pathological Physiology, Centre of Experimental Medicine, Slovak Academy of Sciences, 813 71 Bratislava, Slovakia; andrej.barta@savba.sk (A.B.); martina.cebova@savba.sk (M.C.); 2 Institute of Neuroimunology, Slovak Academy of Sciences, 813 71 Bratislava, Slovakia; andrej.kovac@savba.sk; 3 Institute of Experimental Physics, Slovak Academy of Sciences, 040 01 Kosice, Slovakia; martina.koneracka@saske.sk (M.K.); vlasta.zavisova@saske.sk (V.Z.); 4 Institute of Pathophysiology, Faculty of Medicine, Comenius University, 811 08 Bratislava, Slovakia

**Keywords:** hypertension, heart, pro(renin) receptor, angiotensin II type 1 receptor, ACE, NADPH oxidase, aliskiren, PLA nanoparticles

## Abstract

We aimed to determine effects of aliskiren, a direct renin inhibitor, loaded onto polymeric nanoparticles on the (pro)renin receptor (*Atp6ap2*), angiotensin II type 1 receptor (*Agtr1*), and angiotensin-converting enzyme (*ACE*) gene expression in the heart of spontaneously hypertensive rats (SHR). Twelve-week-old male SHRs were divided into an untreated group and groups treated with powdered aliskiren or aliskiren-loaded nanoparticles (25 mg/kg/day). After three weeks, the accumulation of aliskiren, distribution of polymeric nanoparticles, gene expression of *Atp6ap2* and *Agtr1* receptors and *ACE*, and protein expression of NADPH oxidase along with the conjugated diene (CD) concentration were analyzed. The accumulation of aliskiren in the heart was higher in the aliskiren-loaded nanoparticle group than in the powdered group. The fluorescent signals of nanoparticles were visible in cardiomyocytes, vessel walls, and erythrocytes. Aliskiren-loaded nanoparticles decreased the gene expression of *Atp6ap2* and *ACE*, while not affecting *Agtr1*. Both forms of aliskiren decreased the protein expression of NADPH oxidase, with a more pronounced effect observed in the aliskiren-loaded nanoparticle group. CD concentration was decreased only in the aliskiren-loaded nanoparticle group. We hypothesize that aliskiren-loaded nanoparticle-mediated downregulation of *Atp6ap2* and *ACE* may contribute to a decrease in ROS generation with beneficial effects in the heart. Moreover, polymeric nanoparticles may represent a promising tool for targeted delivery of aliskiren.

## 1. Introduction

Blocking the renin–angiotensin–aldosterone system (RAAS) with angiotensin-converting enzyme (ACE) inhibitors and angiotensin II type 1 receptor blockers decreases high blood pressure and reduces morbidity and mortality in patients with chronic heart failure and chronic kidney disease [1,2]. Although these drugs are highly beneficial, they may not sufficiently inhibit the RAAS. The disadvantage is that ACE inhibitors and angiotensin II type 1 receptor blockers stimulate renin secretion by interrupting angiotensin II feedback inhibition [3]. With growing evidence regarding the direct renin inhibitor aliskiren, additional combined strategies involving renin inhibition have been suggested. Recently, the potential added value of aliskiren in combination with other RAAS modulators, but also as a monotherapy, has been extensively studied [2,3,4].

Aliskiren binds specifically to the S3bp binding site of renin that catalyzes the rate-limiting step of the RAAS [5,6], thus preventing the conversion of angiotensinogen to angiotensin I, leading to decreased formation of angiotensin II primarily through ACE. Subsequently, reduced vasoconstriction, aldosterone secretion, and catecholamine release lead to decreased blood pressure [7]. However, aliskiren has many beneficial effects in addition to its ability to reduce blood pressure, including anti-atherosclerotic, cardioprotective, and renoprotective effects. These beneficial properties of aliskiren are also based on its ability to reduce reactive oxygen species (ROS) production [8,9,10].

Aliskiren has been documented to decrease (pro)renin receptor expression in smooth muscle cells [11], in endothelial cells [12], and in cardiomyocytes [13,14]. (Pro)renin receptor comprises 350 amino acid residues and is encoded by the *Atp6ap2* gene located on the X chromosome at locus p11.4 (NCBI gene ID:10159) [12,15]. It can activate (pro)renin, enhance its activity, and promote angiotensin II formation. In the heart, the (pro)renin receptor may also stimulate electrical alterations and atrial structural remodeling [16]. Moreover, multiple regression analyses showed a significant association with a large coefficient between the arterial mRNA level of the (pro)renin receptor and the arterial mRNA level of *ACE* in kidney failure patients [17]. Aliskiren has been also documented to downregulate hepatic *ACE* mRNA in isolated cirrhotic liver [18] and angiotensin II type 1 receptor in ovariectomized mice [19]. Downregulation or blockade of the angiotensin II type 1 receptor and ACE has been shown to reduce the activation of NADPH oxidases and mitochondrial respiratory activity [20,21,22]. Therefore, it was hypothesized that in addition to the inhibition of renin and angiotensin II production, aliskiren-dependent downregulation of selected components within the RAAS may reduce the expression/activity of NADPH oxidase, thereby decreasing the generation of ROS.

However, the relatively high dose of aliskiren (300 mg) needed for compensation of low bioavailability (2.6–5.0%) and ensuring beneficial effects of this drug may generate different adverse effects [23]. Administration of renin inhibitors can cause high blood potassium levels in diabetic patients, low blood pressure in volume-depleted patients, and angioedema [24]. Currently, different carrier systems such as liposomes, microemulsions, and nanoparticles have been proposed for the protection and effective targeted delivery of different drugs [25]. Among these, biopolymeric nanoparticles represent a promising strategy for minimizing side effects while maximizing drug bioavailability and effectiveness [26].

The aim of our study was to analyze the effect of aliskiren-loaded polylactide acid (PLA) nanoparticles on the (pro)renin receptor (*Atp6ap2*), angiotensin II type 1 receptor (*Agtr1*), and *ACE* gene expression in the hearts of spontaneously hypertensive rats. The protein expression of NADPH oxidase and concentration of conjugated diene (CD) were also studied.

## 2. Results

### 2.1. Weight Parameters

In the control group the body weight was 317 ± 8 g, the heart weight was 1.15 ± 0.04 g, and the HW/BW ratio was 3.63 ± 0.09 × 10^3^. There were no significant changes in any parameters within the groups.

### 2.2. Distribution of Polymeric Nanoparticles in the Heart

**Figure 1:**
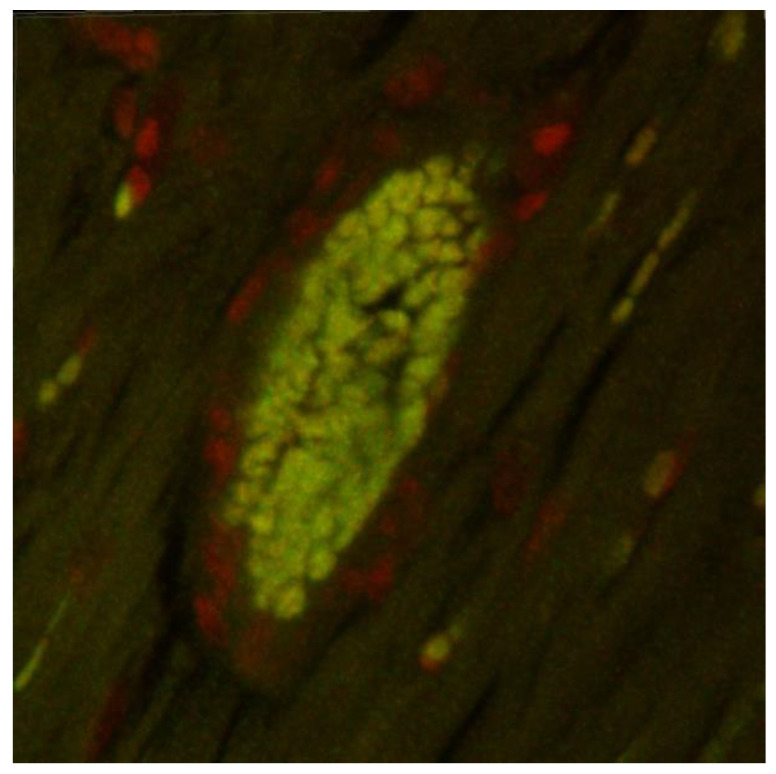
Myocardium with coronary artery—3D reconstruction: coumarin-labeled nanoparticles—green fluorescence, cell nuclei—red fluorescence.

Figure 1 shows a myocardium with a coronary artery in a 3D reconstruction 24 h after the administration of coumarin-labeled nanoparticles. Coumarin-labeled nanoparticles, visible in green fluorescence, were evident in cardiomyocytes and vessel walls, and the strongest signal was in erythrocytes.

### 2.3. Quantitative Analysis of Aliskiren in the Heart

**Figure 2:**
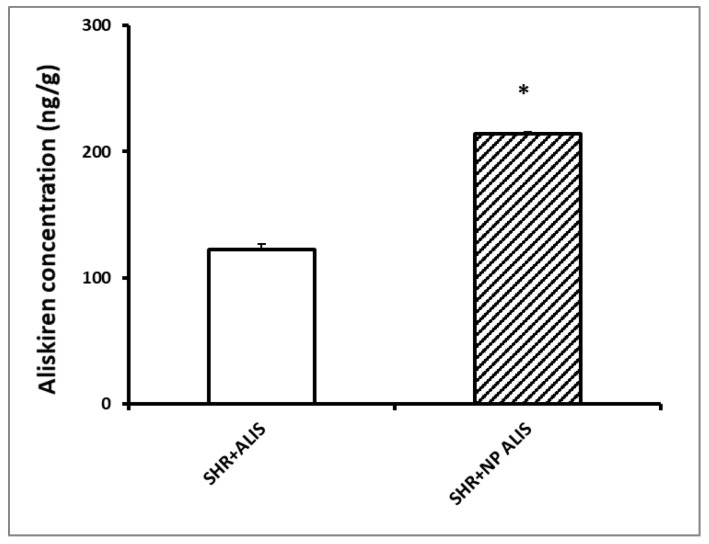
Accumulation of aliskiren in the heart. Control group—SHR, powdered aliskiren group—SHR+ALIS, aliskiren-loaded nanoparticle group—SHR+NP ALIS, * *p* < 0.05 vs. SHR+ALIS.

After three weeks of treatment, accumulation of aliskiren in the heart was higher in the aliskiren-loaded nanoparticle group than in the powdered aliskiren group (Figure 2). The content of aliskiren in the control SHR group was equal to zero.

### 2.4. Profiling of Gene Expression

**Figure 3:**
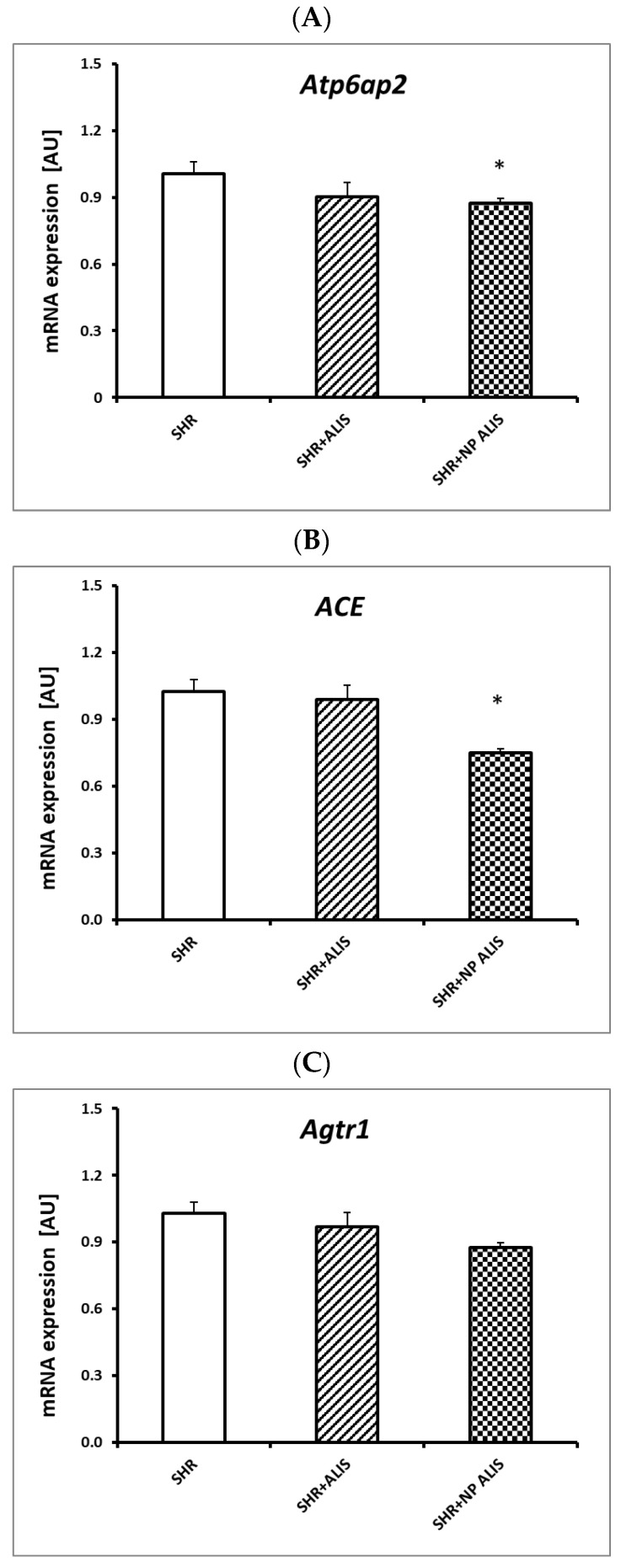
Atp6ap2 (**A**), ACE (**B**), and agtr1 (**C**) gene expression in the heart of control group—SHR, powdered aliskiren group—SHR+ALIS, and aliskiren-loaded nanoparticle group—SHR+NP ALIS, * *p* < 0.05 vs. SHR.

Aliskiren-loaded nanoparticles significantly decreased the gene expression of *Atp6ap2* and *ACE*, while not affecting *Agtr1*. The powdered form of aliskiren did not affect any gene expression studied (Figure 3A–C).

### 2.5. Protein Expression of NADPH Oxidase and Concentration of Conjugated Diene

**Figure 4:**
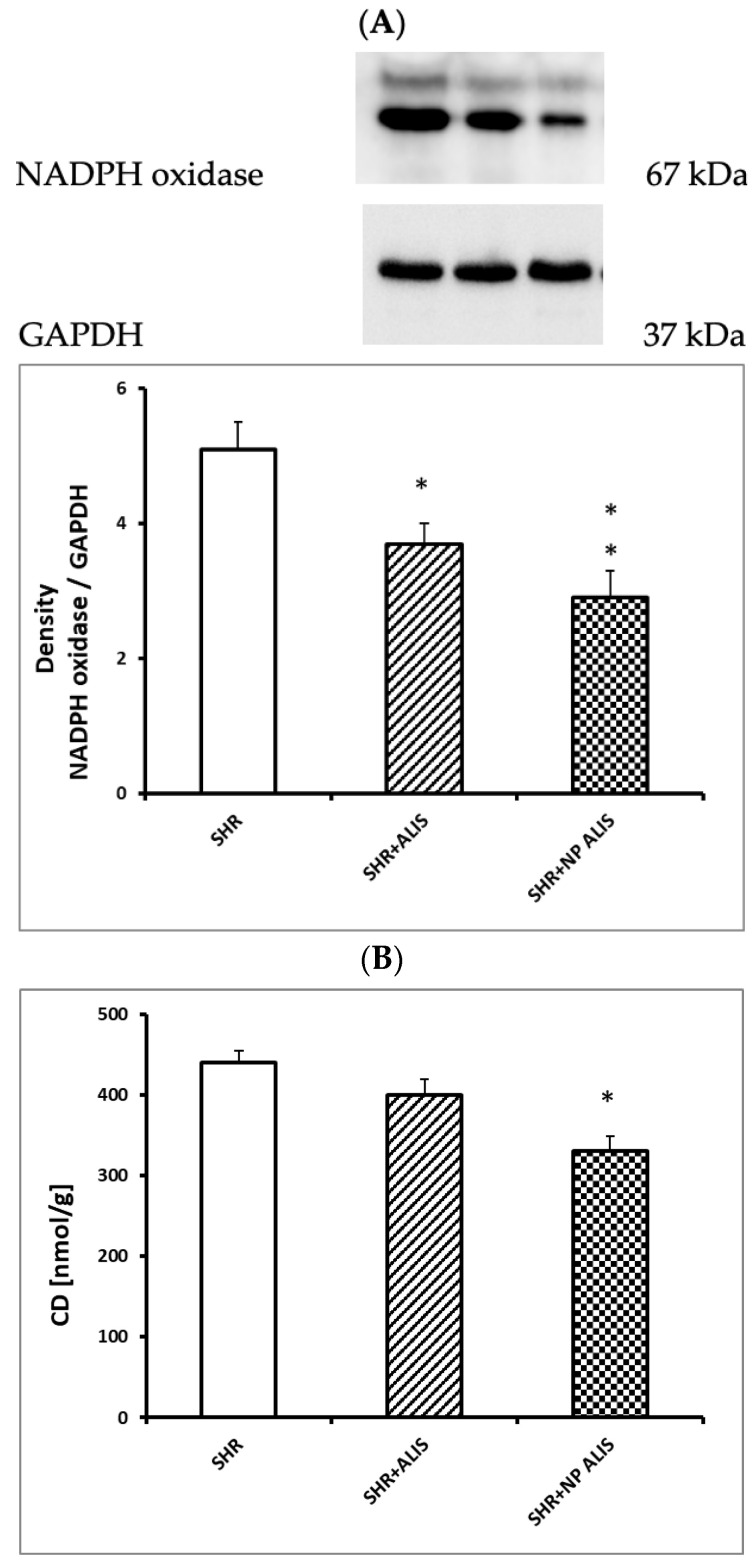
NADPH oxidase protein expression (**A**) and conjugated diene—CD concentration (**B**) in the heart of control group—SHR, powdered aliskiren group—SHR+ALIS, and aliskiren-loaded nanoparticle group—SHR+NP ALIS, * *p* < 0.05 vs. SHR, ** *p* < 0.01 vs. SHR.

Both forms of aliskiren decreased the protein expression of NADPH oxidase, with a more pronounced effect in the aliskiren-loaded nanoparticle group (Figure 4A). CD concentration was decreased significantly only in the aliskiren-loaded nanoparticle group (Figure 4B).

## 3. Discussion

The RAAS continues to be of interest in the quest for new targets in the treatment of cardiovascular and renal diseases as new components, processes, and pathways are discovered. We have documented for the first time that three weeks of aliskiren-loaded polymeric nanoparticle treatment decreased the gene expression of *Atp6ap2* and *ACE* but did not affect *Agtr1* in the heart of spontaneously hypertensive rats. The powdered form of aliskiren did not affect the expression of any of the genes studied. Both forms of aliskiren decreased the protein expression of NADPH oxidase, with a more pronounced effect observed after the treatment with aliskiren-loaded nanoparticles.

The relevance of the (pro)renin receptor in cardiovascular diseases has been discussed since the (pro)renin receptor was discovered [27]. Independent of angiotensin II, activation of the (pro)renin receptor further stimulates intracellular signals, at least linked to oxidative stress and fibrotic processes. (Pro)renin receptor gene delivery by adenovirus-mediated gene transfer into the heart led to angiotensin II-independent activation of extracellular-signal-regulated kinase1/2 phosphorylation (ERK1/2), expression of transforming growth factor-β1 (TGF-β1), and connective tissue growth factor genes associated with myocardial fibrosis [28]. Moreover, an association between (pro)renin gene polymorphisms and cardiovascular disease has been reported [29]. Receptor-bound renin has been shown to also induce a series of intracellular effects that are distinct from the generation of angiotensin II [30].

Similarly, ACE, in addition to hypertension, is involved in several other renal diseases, such as diabetic kidney disease and acute kidney injury, even when blood pressure is normal. In addition, several studies have suggested that ACE might mediate at least part of its effect through mechanisms that are independent of angiotensin I conversion into angiotensin II and involve other substrates such as Ang-(1-7), N-acetyl-seryl-aspartyl-lysyl-proline, or bradykinin [31]. Thus, the downregulation of these RAAS components may have different cardioprotective and renoprotective effects. Aliskiren-loaded nanoparticles were able to downregulate the expression of *Atp6ap2* and *ACE* under our experimental conditions.

Aliskiren, a direct renin inhibitor, has been documented to decrease (pro)renin receptor expression in smooth muscle, in endothelial cells, and in cardiomyocytes [11,12,13,14]. However, in our experimental study, the treatment of SHRs with powdered aliskiren at a dose of 25 mg/kg/day for three weeks did not show any significant effect on the downregulation of these RAAS components. In contrast, loading aliskiren to the PLA nanoparticles decreased the expression of *Atp6ap2* and *ACE* in the heart. We hypothesized that the PLA nanoparticles would increase aliskiren’s bioavailability and thus the effectiveness of aliskiren in the heart. Indeed, quantitative analysis revealed higher accumulation of aliskiren in the aliskiren-loaded nanoparticle group than in the powdered aliskiren group. Ferri et al. [11] also documented a decrease in (pro)renin receptor expression in smooth muscle cells following aliskiren administration. The lower levels of the (pro)renin receptor in their experiment were associated with reduced expression of TGF-β, plasminogen activator inhibitor-1, and type I collagen mRNA. Similarly, our previous study demonstrated a decrease in collagen content and cross-sectional area in the aorta after treatment with aliskiren-loaded nanoparticles [32]. In the TGR(m(Ren2)-27) model of diabetic rats, aliskiren-mediated reduction in cardiac pro(renin) receptor expression was associated with improved cardiac function and structure [14]. Cao et al. [33] demonstrated that the (pro)renin receptor induces myocardial fibrosis and deteriorates cardiac function via ROS generation from the (pro)renin receptor–ERK1/2-NOX4 pathway during alcoholic cardiomyopathy development. In streptozotocin-induced diabetic mice, aliskiren protected the heart from increased expression of the (pro)renin receptor simultaneously with decreased oxidative stress [13]. Moreover, (pro)renin receptor expression and local angiotensin II production were upregulated in skeletal muscle from post-infarct heart failure mice and were attenuated in aliskiren-treated mice, in tandem with a decrease in NADPH oxidase activity and superoxide generation [34].

In agreement with these studies, under our experimental conditions, both forms of aliskiren decreased the protein expression of NADPH oxidase, with a more pronounced effect in the aliskiren-loaded nanoparticle group. In addition, Peng et al. [35] showed that in neuro-2A human cells, (pro)renin receptor overexpression increased ROS production, NADPH oxidase activity, and NADPH oxidase isoform 2 and 4 mRNA expression. These effects were prevented by mitogen-activated protein kinase/extracellular signal-regulated kinase 1 and 2 (MAPK/ERK1/2) inhibition and phosphoinositide 3 kinase/Akt (IP3/Akt) inhibition, but not by losartan. These results indicate that the (pro)renin receptor regulates NADPH oxidase activity and ROS formation through angiotensin II-independent pathways and are in good agreement with our study.

In our experiment, in addition to the reduction in the protein expression of NADPH oxidase, a significant decrease in the concentration of conjugated dienes in the aliskiren-loaded nanoparticle group was demonstrated. This may indicate a reduction in lipid peroxidation in this particular group of SHR. Similarly, aliskiren pretreatment significantly reduced malondialdehyde concentration in the heart and improved the serum levels of lactate dehydrogenase, total cholesterol, triglycerides, and low-density lipoprotein in doxorubicin-treated rats [36]. Aliskiren treatment also resulted in a significant decrease in the levels of lipid peroxides in bleomycin-induced pulmonary fibrosis in Wistar rats [37].

Our study did not show any changes in *Agtr1* expression after three weeks of aliskiren treatment. This means that the gene expression of *Agtr1* was not responsible for the downregulation of NADPH oxidase, and probably downregulation of the (pro)renin receptor and *ACE* at least contributed to the reduction of both NADPH oxidase expression and lipid peroxidation. Furthermore, the PLA nanoparticles increased the bioavailability of aliskiren, leading to the significant decrease in *Atp6ap2* and *ACE* gene expression in the heart in our experimental study. The effect of empty polymeric nanoparticles on the parameters measured in our study was determined as well. However, empty nanoparticles did not affect any parameter. PLA nanoparticles also increased the bioavailability of aliskiren in the kidney, with aliskiren incorporation in the kidney being higher than in the heart.

Similarly, in our previous experiments, only aliskiren-loaded nanoparticles improved endothelium-dependent relaxation. Moreover, aliskiren-loaded nanoparticles reduced blood pressure more significantly than powdered aliskiren, suggesting better drug efficacy [26,32]. In addition, Nakano et al. [38] developed bioabsorbable poly-lactic/glycolic acid (PLGA) nanoparticles loading irbesartan, an angiotensin II type 1 receptor blocker with a peroxisome proliferator-activated receptor (PPAR)γ agonistic properties (irbesartan-NP). In a mouse model of myocardial ischemia–reperfusion injury, they demonstrated that a single intravenous treatment at the time of reperfusion with irbesartan-NP, but not with irbesartan solution, inhibited the recruitment of inflammatory monocytes to the heart, reduced infarct size via PPARγ-dependent anti-inflammatory mechanisms, and improved heart remodeling. Actually, different types of nanoparticles, such as chitosan, polymeric, and nanofiber, have been examined in RAAS-related studies, especially involving hypertension and cardiovascular disease [39]. Among these, polymeric nanoparticles may represent a promising tool with a different range of use.

## 4. Materials and Methods

### 4.1. Chemicals

Most of the chemicals and reagents were obtained from Sigma-Aldrich (Saint-Louis, MO, USA); otherwise, the company is indicated.

### 4.2. Polymeric Nanoparticles: Preparation and Characterization

The preparation and physicochemical characterization of the nanoparticles have been described in detail in our previous studies [32,40]. Briefly, blank nanoparticles (PLA NPs) and aliskiren-loaded polymeric nanoparticles (PLA Alis NPs) were prepared using a modified nanoprecipitation method. Both PLA NPs and PLA Alis NPs were characterized with respect to size, morphology, zeta potential, drug loading, and encapsulation efficiency. After studying the effects of various processing parameters on mean particle size and drug content, PLA Alis NPs with theoretical aliskiren loading of 5 mg Alis/100 mg NPs were selected for the subsequent in vivo experiments.

**Figure 5:**
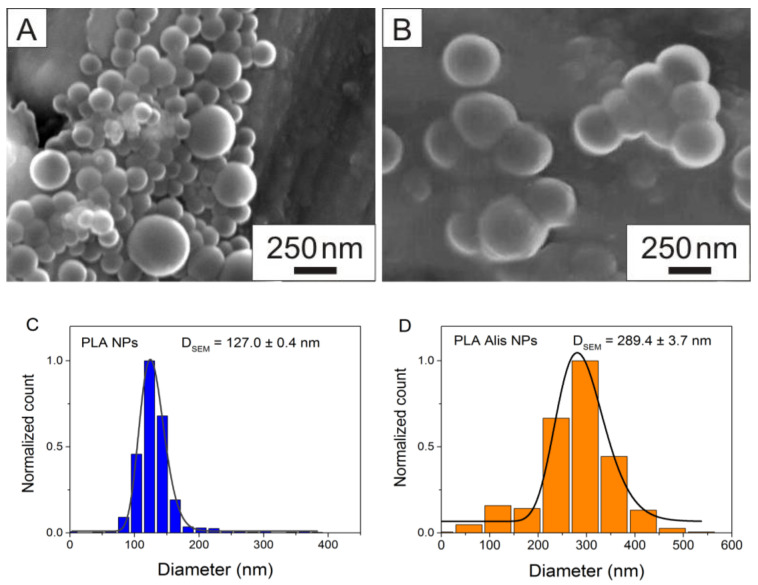
SEM images of blank polymeric nanoparticles—PLA NPs (**A**) and aliskiren-loaded polymeric nanoparticles—PLA Alis NPs (**B**) and corresponding size distributions (**C**,**D**), D—diameter.

In this study, three different methods were used to perform the complex physicochemical characterization of the samples published in the previous study [28]. Scanning electron microscopy (SEM) was used to study the morphologies of PLA NPs and PLA Alis NPs, as well. Figure 5 shows an SEM image of PLA NPs (A) and aliskiren-loaded polymer NPs (B) and their corresponding size distributions (C and D). The nanoparticles appeared to be spherical in shape, and the surface was smooth. As can be seen in the figures, most PLA nanoparticles were smaller than the aliskiren-loaded nanoparticles. This is also evidenced by the size distributions obtained manually from 20 images and 1300 particles. The mean particle sizes of PLA NPs and PLA Alis NPs, determined from the log-normal fit of the histogram, were found to be 127.0 ± 0.4 nm and 289.4 ± 3.7 nm, respectively, confirming successful aliskiren encapsulation into PLA nanoparticles. The SEM provides visual and descriptive information, a real overview about the nanoparticle population. However, this information should be compared by a complementary method such as the differential centrifugal sedimentation technique (DCS) to obtain an overall picture of the particle size.

**Figure 6:**
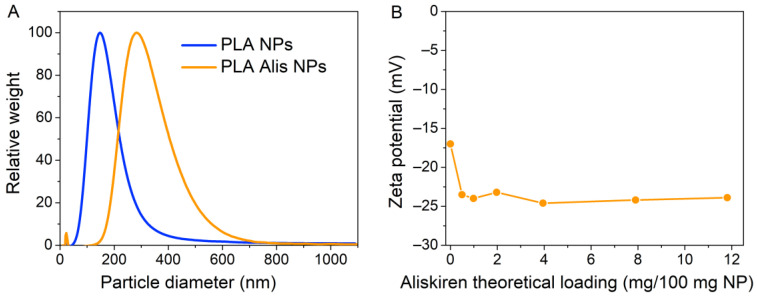
Size distribution of blank polymeric nanoparticles—PLA NPs and aliskiren-loaded polymeric nanoparticles—PLA Alis NPs as measured by differential centrifugal sedimentation—DCS (**A**); zeta potential of nanoparticle as a function of aliskiren concentration (**B**).

DCS is the next sizing technique utilized for particle size analysis, which separates particles by size using centrifugal sedimentation in a liquid medium. Despite the different physical principles upon which DCS is based, the obtained results of mean diameters of the PLA NPs (D_DCS_ = 167 nm) and PLA Alis NPs (D_DCS_ = 300 nm) are comparable to those obtained by SEM (see Figure 6A) and to outcomes measured from DLS results presented in the previous study [32].

The third method is laser Doppler microelectrophoresis, which is measured using a Malvern Zetasizer Nano ZS and results in zeta potential. The zeta potential is the electrostatic potential on the slip surface of a particle dispersed in a liquid environment and helps to characterize the surface of a colloidal drug carrier system, which in our case is PLA Alis NPs. Figure 6B shows the influence of the aliskiren concentration used to prepare nanoparticles on the zeta potential of the final nanoparticle suspension. As shown in the figure, the increased loading aliskiren concentration caused the zeta potential to decrease to a plateau at −24 mV, suggesting the presence at least part of the drug on the nanoparticle surface. These results correlated well with aliskiren release experiments at pH 2, 4.5, and 7.4, as reported in the previous study [32].

### 4.3. Animals and Treatment

Procedures and experimental protocols were performed in accordance with institutional guidelines and were approved by the State Veterinary and Food Administration of the Slovak Republic (No. 3259/12-221) and by the Ethical Committee of the Centre of Experimental Medicine, Slovak Academy of Sciences, according to the European Convention for the Protection of Vertebrate Animals used for Experimental and other Scientific Purposes, Directive 2010/63/EU of the European Parliament. Male, 12-week-old spontaneously hypertensive rats were divided into an untreated group (SHR) and groups treated with powdered aliskiren at a dose of 25 mg/kg/day (SHR+ALIS) or nanoparticle-loaded aliskiren at a similar dose (SHR+NP ALIS). Each group consisted of six animals. The treatment was administered via gavage for 3 weeks. All animals were housed at a temperature of 22–24 °C, constant humidity (45–65%), under a 12 h light–12 h dark cycle, with free access to standard laboratory rat chow and drinking water.

At the end of the treatment, the animals were sacrificed, and their body weight (BW) and heart weight (HW) were determined. Relative heart weight was calculated as the HW/BW ratio. Samples of the left ventricle were used for quantitative and morphological analyses of powdered aliskiren and aliskiren-loaded nanoparticles and the gene expression of atp6ap2, agtr1, and ACE. The protein expression of NADPH oxidase and CD concentration were also determined in the left ventricle of the heart.

### 4.4. Distribution of Polymeric Nanoparticles in the Heart

Fluorescent dye (coumarin-6)-loaded nanoparticles were used to determine the PLA nanoparticle distribution in the rat body. Briefly, two male 12-week-old SHRs were gavaged once with fluorescent nanoparticles (500 mg/kg). After 24 h, the rats were sacrificed, and tissue samples were fixed by immersion in 4% neutral buffered formaldehyde and after 24 h embedded in paraffin using standard techniques. Paraffin blocks were cut into 50 μm-thick sections. After deparaffinization in xylene and rehydration in graded ethanol, the nuclei were counterstained with propidium iodide. Subsequently, a confocal microscope (NIKON Ti-E C2+, Nikon Europe B.V., Amstelveen, The Netherlands) equipped with epifluorescence optics and a combination of filters was used to visualize and digitize images of the heart specimens.

### 4.5. Quantitative Analysis of Aliskiren in the Heart

Quantitative analysis of aliskiren in the heart was carried out by ultra-performance liquid chromatography–tandem mass spectrometry using an ACQUITY ultra-performance liquid chromatography UPLC system (Waters Corporation, Milford, MA, USA) coupled to a Quattro Premier XE triple-quadrupole mass spectrometer (Waters Corporation, Milford, MA). Chromatographic separation was performed in reversed-phase mode on an ACQUITY UPLC BEH C8 1.7 µm (2.1 × 50 mm) column maintained at 35 °C. The flow rate was 0.5 mL/min^−1^. Mobile phase A consisted of 0.1% formic acid in water, and mobile phase B consisted of 100% acetonitrile. The following elution gradient was used: 10% B in 0–0.4 min, 10–90% B in 0.4–2 min, 90% B in 2.0–2.5 min, and 90–10% B in 2.5–3.0 min. The injection volume was 5 µL. Nevirapine was used as an internal standard. The mass spectrometer was operated in MRM mode (transitions *m*/*z* 552.3–436.4, 552.3–117 for aliskiren; *m*/*z* 267.4–226.3 for nevirapine). Samples were analyzed in positive electrospray mode; the capillary voltage was 3.00 kV. The source and desolvation temperatures were 120 °C and 450 °C, respectively, and the flow rate of desolvation gas was 600 L/hour. Acquisition and evaluation of acquired data were carried out using the MassLynx 4.1. software. Samples were prepared by homogenization of tissue in 100 mM ammonium carbonate buffer (pH 9.5). Supernatant was transferred to another tube and extracted using tert-butyl methyl ether. The capped tubes were centrifuged at 5000× *g* for 10 min. The tert-butyl methyl ether layer was evaporated in a Savant Speed vac (Thermo Fischer Scientific, Waltham, MA, USA), and samples were reconstituted in 100 μL of 10% acetonitrile/MPW with 0.1% formic acid, transferred into sample vials, and analyzed.

### 4.6. Profiling of Gene Expression

Gene expression profiling was performed as previously described [41,42]. Briefly, tissue samples were homogenized using MagnaLyser Green Beads (Roche, Basel, Switzerland), and total RNA was extracted using a Genelute Mammalian Total RNA Miniprep Kit (Sigma-Aldrich, St Louis, MO, USA) according to the manufacturer’s instructions. The quantity and purity of RNA were checked by a NanoDrop ND 1000 spectrophotometer (NanoDrop products, Wilmington, DE, USA). For removal of residual DNA, an RNase-Free DNase Set (Qiagen, Hilden, Germany) was used. Two µg of total RNA was transcribed to cDNA using a High-Capacity cDNA Reverse Transcription Kit (Life Technologies, Carlsbad, CA, USA) with random primers, according to manufacturer’s instructions, and diluted aliquots of synthesized first-strand cDNA were stored at −20 °C. Measurement of gene expression was performed using a HOT FIREPol^®^ Probe qPCR Mix Plus (SolisBioDyne, Tartu, Estonia) and TaqMan^®^ Gene Expression Assays (Life Technologies, Carlsbad, CA, USA) on a LightCycler^®^ 480 System (Roche, Basel, Switzerland). The following TaqMan^®^ Gene Expression Assays were used: *Atp6ap2* Rn01430718_m1, *Agtr1A* Rn01435427_m1, *ACE* Rn00561094_m1, *Gapdh* Rn01775763_g1, and *Hprt1* Rn01527840_m1. Thermocycling conditions were set as recommended for TaqMan^®^ Gene Expression Assays: initial denaturation (95 °C for 10 min), 45 cycles of denaturation (95 °C for 15 s), and annealing/elongation (60 °C for 1 min). Baseline estimation error-free analysis was performed in LinRegPCR (version 2013.0) on exported raw data, which set a fluorescence threshold for the determination of the Ct value. The data were normalized to the two most stable references concerning the heart (*Gapdh* and *Hprt1*), which were selected as previously described [43].

### 4.7. Protein Expression of NADPH Oxidase and Concentration of Conjugated Diene

Samples of the left ventricle were homogenized. Western blot analysis was performed as described elsewhere [44]. The membranes were incubated overnight with primary polyclonal rabbit anti-NADPH oxidase 4 (1:2000, Abcam, ab154244) and anti-GAPDH (1:5000, Abcam, ab201822) as loading controls. The antibodies were detected using a secondary peroxidase-conjugated goat anti-rabbit antibody (1:5000, Abcam, ab97051) at room temperature for 2 h. The intensity of the bands was visualized using an enhanced chemiluminescence system (ECL, Amersham, UK), quantified using a ChemiDocTM Touch Imagine System (Image LabTM Touch software, version 5.2 build 14, 11 September 2014, BioRad, Hercules, CA, USA), and normalized to GAPDH bands.

The concentration of CD was measured in the lipid extracts of the left ventricle homogenates as previously described [45]. Briefly, after chloroform evaporation under an inert atmosphere and the addition of cyclohexane, the CD concentration was determined spectrophotometrically (λ = 233 nm, GBC 911A, Bio-Rad Laboratories GesmbH, Wien, Austria).

### 4.8. Statistics

The results are expressed as mean ± S.E.M. One–way ANOVA and the Bonferroni post hoc test were used for statistical analysis. Values were considered significant with a probability value *p* < 0.05. *p* values were multiplicity-adjusted.

## 5. Conclusions

In conclusion, PLA nanoparticles have the potential to increase bioavailability of aliskiren in the heart, guaranteeing its beneficial effect. In our experimental conditions, three weeks of aliskiren-loaded polymeric nanoparticle treatment decreased the gene expression of *Atp6ap2* and *ACE*. The powdered form of aliskiren did not affect the expression of these genes. Both forms of aliskiren decreased the protein expression of NADPH oxidase, with a more pronounced effect observed after treatment with aliskiren-loaded polymeric nanoparticles. The novelty of our study is therefore (i) the finding that polymeric nanoparticles extended the bioavailability of aliskiren, which was thus able to reduce the gene expression of *Atp6ap2* and *ACE*, and that (ii), this reduction contributed to the decrease in NADPH oxidase protein expression. In addition, (iii) the downregulation of NADPH oxidase protein expression further contributed to the decrease in the concentration of conjugated dienes and potentially to reduced lipid peroxidation, which was observed only in rats treated with aliskiren-loaded polymeric nanoparticles.

## Data Availability

Data supporting reported results can be found in archived datasets of the Department of Neuro-Cardiovascular Interactions, Institute of Normal and Pathological Physiology, Centre of Experimental Medicine, Slovak Academy of Sciences.
